# No accelerated arterial aging in relatively young women after preeclampsia as compared to normotensive pregnancy

**DOI:** 10.3389/fcvm.2022.911603

**Published:** 2022-07-28

**Authors:** Emma B. N. J. Janssen, Mieke C. E. Hooijschuur, Veronica A. Lopes van Balen, Erjona Morina-Shijaku, Julia. J. Spaan, Eva G. Mulder, Arnold P. Hoeks, Koen D. Reesink, Sander M. J. van Kuijk, Arnoud van't Hof, Bas C. T. van Bussel, Marc E. A. Spaanderman, Chahinda Ghossein-Doha

**Affiliations:** ^1^Department of Obstetrics and Gynaecology, Maastricht University Medical Centre+ (MUMC+), Maastricht, Netherlands; ^2^Cardiovascular Research Institute Maastricht (CARIM), Maastricht University, Maastricht, Netherlands; ^3^Department of Biomedical Engineering, MUMC+, Maastricht, Netherlands; ^4^Department of Clinical Epidemiology and Medical Technology Assessment, MUMC+, Maastricht, Netherlands; ^5^Department of Cardiology, MUMC+, Maastricht, Netherlands; ^6^Department of Cardiology, Zuyderland Medical Centre, Heerlen, Netherlands; ^7^Department of Intensive Care Medicine, MUMC+, Maastricht, Netherlands; ^8^Care and Public Health Research Institute (CAPHRI), Maastricht University, Maastricht, Netherlands; ^9^Department of Obstetrics and Gynaecology, Radboud University Medical Centre, Nijmegen, Netherlands

**Keywords:** aging, preeclampsia, cardiovascular, flow-mediated dilation (FMD), arterial function, endothelial (dys)function, hypertensive pregnancy

## Abstract

**Introduction:**

Preeclampsia, an endothelial disorder of pregnancy, predisposes to remote cardiovascular diseases (CVD). Whether there is an accelerated effect of aging on endothelial decline in former preeclamptic women is unknown. We investigated if the arterial aging regarding endothelial-dependent and -independent vascular function is more pronounced in women with a history of preeclampsia as compared to women with a history of solely normotensive gestation(s).

**Methods:**

Data was used from the Queen of Hearts study (ClinicalTrials.gov Identifier NCT02347540); a large cross-sectional study on early detection of cardiovascular disease among young women (≥18 years) with a history of preeclampsia and a control group of low-risk healthy women with a history of uncomplicated pregnancies. Brachial artery flow-mediated dilation (FMD; absolute, relative and allometric) and sublingually administered nitroglycerine-mediated dilation (NGMD; absolute and relative) were measured using ultrasound. Cross-sectional associations of age with FMD and NGMD were investigated by linear regression. Models were adjusted for body mass index, smoking, antihypertensive drug use, mean arterial pressure, fasting glucose, menopausal state, family history of CVD and stress stimulus during measurement. Effect modification by preeclampsia was investigated by including an interaction term between preeclampsia and age in regression models.

**Results:**

Of the 1,217 included women (age range 22–62 years), 66.0% had a history of preeclampsia and 34.0% of normotensive pregnancy. Advancing age was associated with a decrease in relative FMD and NGMD (unadjusted regression coefficient: FMD: −0.48%/10 years (95% CI:−0.65 to −0.30%/10 years), NGMD: −1.13%/10 years (−1.49 to −0.77%/10 years)) and increase in brachial artery diameter [regression coefficient = 0.16 mm/10 years (95% CI 0.13 to 0.19 mm/10 years)]. Similar results were found when evaluating FMD and NGMD as absolute increase or allometrically, and after confounder adjustments. These age-related change were comparable in former preeclamptic women and controls (*p*-values interaction ≥0.372). Preeclampsia itself was independently associated with consistently smaller brachial artery diameter, but not with FMD and NGMD.

**Conclusion:**

In young- to middle-aged women, vascular aging in terms of FMD and NGMD was not accelerated in women after preeclampsia compared to normotensive pregnancies, even though former preeclamptic women consistently have smaller brachial arteries.

## Introduction

Arterial aging is a physiological process that develops gradually over time and increases the risk of cardiovascular diseases (CVD). With advancing age, several structural and functional arterial changes contribute to this increased cardiovascular (CV) risk (1–3). Age-related vascular dysfunction is characterized by a decline in endothelial function involving impaired vasodilatory capacity of the blood vessel.

Besides age, vascular dysfunction may be accelerated by both conventional and sex-specific CV risk factors (4, 5). Preeclampsia (PE), a hypertensive vascular complication of pregnancy, is associated with impaired endothelial function, both during pregnancy and in the first years after delivery (6, 7). Endothelial dysfunction during or after PE might contribute to the subsequently observed two- to seven-fold increased risk of CVD among these women (8, 9). On top of that, vascular aging might further be accelerated in former preeclamptic women since conventional risk factors, especially increased blood pressure, are highly prevalent after PE (10).

The major mediator of vasodilatory capacity of arteries is endogenous nitric oxide (NO) release by the endothelium, which relaxes vascular smooth muscles (11, 12) resulting in flow-mediated vasodilation in healthy conditions. This physiological response is favorable as it keeps local wall shear stress constant (13). Flow-mediated dilation (FMD) measurement of the brachial artery is a non-invasive method to assess endothelial dysfunction by high-resolution ultrasound imaging. Impaired FMD is used as surrogate measure for CV health as it is strongly associated with and predictive of CVD later in life (14–17).

Whether PE modifies the age-related decline in endothelial function is unknown. Therefore, we investigated whether the age-related decline in endothelial-dependent and –independent vasodilatory function is more pronounced in women with a history of PE as compared to women with normotensive pregnancies, independent of conventional CV risk factors.

## Materials and methods

### Study design and population

This study was part of a large cross-sectional study aimed at investigating subclinical CVD in women (Queen of Hearts study; ClinicalTrials.gov Identifier NCT02347540) and was approved by the Medical Ethics Committee of the Maastricht University Medical Center (METC azM/UM 14-2-20136 NL47252.068.14). All participating women provided written informed consent. Procedures were in conformity with institutional guidelines and adhered to the principles of the Declaration of Helsinki.

We included women aged ≥18 years with a history of PE and a control group of women who had normotensive pregnancies. Women were included within a postpartum interval of 0.5–30 years, which was based on delivery of their first (complicated) pregnancy. Women who participated between December 2014 and October 2019 were included in the current study. Women with a history of hypertension, autoimmune disease, or kidney disease prior to their first pregnancy were excluded.

PE was defined as new-onset hypertension (i.e. systolic blood pressure (SBP) ≥140 mmHg and/or diastolic blood pressure (DBP) ≥90 mmHg) along with proteinuria (≥300 mg/24 h) after 20 weeks of gestation, or other maternal organ dysfunction (18). Diagnosis before 34 weeks was characterized as early onset PE. Uncomplicated pregnancy was defined as normotensive pregnancy in absence of any placenta-associated disease, including HELLP-syndrome, placental abruption, small for gestational age infancy and/or fetal demise.

### Cardiovascular assessment

Postpartum cardiovascular assessment was performed following a standardized protocol during one morning study visit at the Maastricht University Medical Center (MUMC+), including vascular assessment, physical examination of weight and height, blood pressure measurements, fasting blood collection and (obstetrical) medical history taking. All women were instructed to fast for at least 10 h before the study visit.

Height and weight were measured to calculate body mass index (BMI). SBP, DBP, mean arterial pressure (MAP) and heart rate were measured for 30 min in sitting position by a semiautomatic oscillometric device (Dinamap Vital Signs Monitor 1846, Critikon, Tampa, FL) with a three-minute measurement interval. The median value of these measurements was used for analyses. Hypertension was defined as antihypertensive drug use or SBP ≥140 and/or DBP ≥90 mmHg, as according to European guidelines (19). A positive family history of CVD was defined as a (grand)parent or sibling below the age of 65 years with CVD.

### Measurements of endothelium-dependent and -independent vasodilation

Endothelium-dependent and -independent brachial artery dilation were evaluated by assessing FMD and the effect of a sublingual dose of nitroglycerine (i.e., nitroglycerine-mediated dilation [NGMD]), respectively. FMD and NGMD measurements were performed sequentially under standardized conditions in a temperature-controlled room (±22 °C). Before the measurements, participants rested in supine position on a comfortable bed for at least 15 min. The arm was in an extended position at ±80° from the torso. A rapid inflation and deflation cuff (Hokanson, Bellevue, VA 98005) was positioned around the forearm distal to the olecranon. A multi-frequency linear array probe attached to a high-resolution ultrasound machine (Voluson p6, GE Healthcare) with an operating frequency of five MHz was used to image the brachial artery in the distal third of the upper arm, two to five cm above the antecubital fossa. The probe was fixed during all measurements by a custom-made fixation device made by instrumental services.

For (endothelium-dependent) FMD evaluation, we acquired a 3-min baseline recording of the brachial artery diameter. Thereafter, the forearm cuff was inflated (200 mmHg) for 5 min followed by rapid deflation. The diameter and Doppler spectrum were assessed continuously from 3 min before inflation to 5 min after deflation, but interrupted from 30 s after inflation to 30 s before deflation.

For (endothelium-independent) NGMD evaluation, we also started with a 3-min baseline recording after which a dose of NG (0.4 mg/dose) was administered sublingually. The recording ended 10 min after sublingual administration of NG.

Image analysis of the brachial artery diameter was performed off-line with a custom designed edge-detection and wall-tracking software (13) in Matlab (Matlab R2013b, The Mathworks Inc. Natick, MA), which separates the measurements from the analyses and therefore reduced the risk of bias. Peak diameter was automatically detected, as described previously (13). In a previous pilot, two experienced sonographers performed repeated measurements of FMD in 15 volunteers, to quantify the inter-observer agreement. The corresponding inter-observer intraclass correlation coefficient (ICC) was 0.82, while the intra-observer ICC was 0.83.

### FMD and NGMD outcome measures

FMD and NGMD were expressed both as an absolute and relative (i.e., percentage) increase in brachial artery diameter, which were based on the peak change in diameter with respect to baseline. Baseline diameter during FMD referred to the 3-min period before cuff-release. During NGMD, a single value for baseline was measured at the start of the response. Although the baseline brachial artery diameter was separately measured in FMD- and NGMD-assessment, the variability of these measurements was not statistically significant (paired sample *t*-test; *p* = 0.659).

FMD was also expressed with an allometric scaling to avoid baseline dependency as proposed by Atkinson et al. (20). It aims to compensate for potential differences in vessel diameter. For the allometric FMD, we calculated the regression slope between logarithmically transformed values of both baseline diameter and peak diameter and derived the correct scaling exponent for our dataset (20). A value of 1 is necessary for appropriate use of FMD% (21, 22). The regression slope between the logarithmically transformed values of both baseline diameter and peak diameter yielded a 1.014 scaling exponent, which was used to calculate the allometric scaled FMD.

Besides, we calculated the physiologic dilatory response to stress (i.e., FMD) as proportion of maximal dilatory capacity (i.e., NGMD) by (FMD%^*^100)/NGMD%.

### Statistical analysis

Baseline data are presented as mean and standard deviation in case of normal distribution, otherwise as median and interquartile range [IQR]. Categorical variables are presented as number and percentage within group. To analyze between-group differences in baseline characteristics, we used the independent-samples *t*-test, Mann-Whitney U or Fisher's exact, as appropriate. To ensure no selection bias had occurred due to missing, incomplete or low-quality data on FMD and NGMD, we compared baseline characteristics of women who were excluded to those included in the analyses.

First, differences between age groups (20–29, 30–39, 40–49, ≥50 years) regarding brachial artery diameter, FMD, and NGMD were tested using one-way ANOVA and Kruskal Wallis tests, as appropriate. Subsequently, linear regression analysis was performed to evaluate the association of age with brachial artery diameter, FMD and NGMD, both unadjusted and fully-adjusted for BMI, smoking, antihypertensive drug use, MAP, fasting blood glucose level, menopausal state and a positive family history of CVD. When investigating absolute FMD and NGMD, we additionally adjusted for the baseline diameter. In all regression models on FMD and NGMD, we additionally adjusted for the potential effect of stress stimulus by adjusting for the velocity area under the curve (vAUC) as measured by Doppler during FMD and NGMD assessments, as described previously (13). Effect estimates (β) of the association between age and vascular function were presented per 10 years of advancing age.

Second, the potential interaction effect of age and history of PE was investigated by adding an interaction term between age and history of PE to the linear regression models described above. If the interaction term was not statistically significant (i.e. *p* > 0.10), the interaction was omitted from the model and we only evaluated the effect estimate of PE, while adjusting for age.

Finally, sensitivity analyses were performed to evaluate the association of postpartum interval instead of age and the robustness of our findings following the parameters we used to operationalize FMD and NGMD. We repeated the above analyses 1) by replacing age by postpartum interval, 2) by replacing leading baseline by baseline measured at a single point before response, 3) by replacing relative FMD by normalized relative FMD for stress stimulus (i.e., FMD%/stimulus instead of separate adjustment).

Statistical analyses were performed using the statistical software program IBM SPSS (version 24.0). *P*-values of main effects <0.05 and *p*-values of interactions <0.10 were considered statistically significant.

## Results

### Study population

**Figure 1:**
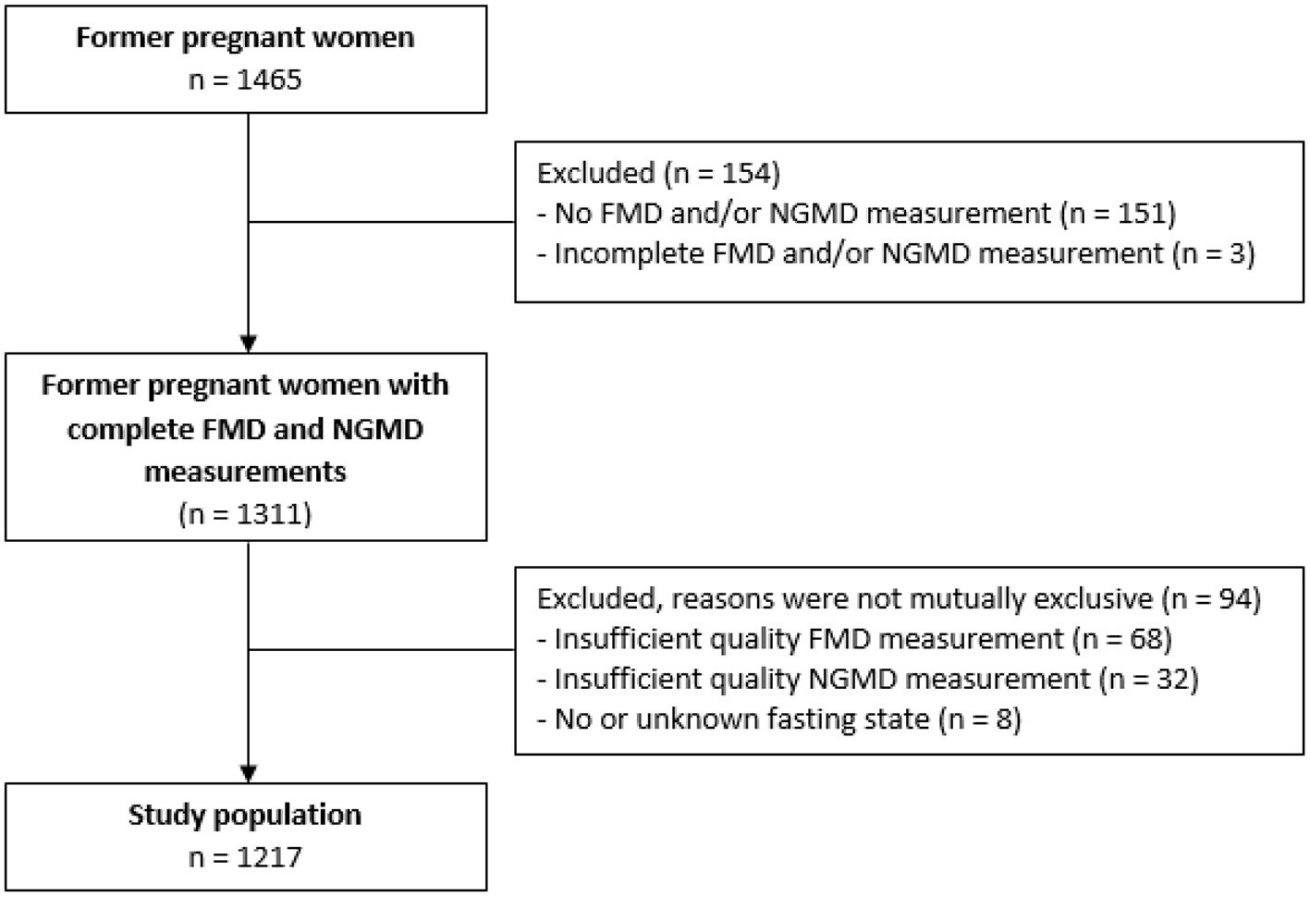
Flowchart of inclusion study population. FMD, flow-mediated-dilation; NGMD, nitroglycerine-mediated dilation.

Of the 1,465 participating women, 248 women were excluded from analysis due to (1) missing-, incomplete or low-quality FMD and NGMD measurements or (2) no or uncertain fasting state before the vascular evaluation (Figure 1). Baseline characteristics of in- and excluded women were comparable except for a higher BMI among those excluded (0.6 kg/m^2^ higher, *p* = 0.018, Supplementary Table 1).

Of the 1,217 eligible participants, 803 (66.0%) had a history of PE and 414 (34.0%) women had a normotensive pregnancy. Baseline characteristics of the total study population and groups are presented in Table 1.

**Table 1:** Baseline characteristics of entire study population and stratified for history of preeclampsia.

	**Total study population (*n* = 1217)**	**Women with history of PE (*n* = 803)**	**Women without history of PE (*n* = 414)**	***p*-value**
Age (years)	40.5 ± 8.6	38.5 ± 7.9	44.5 ± 8.4	*<0.001*
Parity	2 [1–2]	2 [1–2]	2 [2–3]	*<0.001*
Early onset PE^a^	411 (33.8%)	411 (51.2%)	N.A.	N.A.
HELLP-syndrome	567 (46.6%)	567 (70.6%)	N.A.	N.A.
Months postpartum	104 [28–198]	71 [17–155]	180 [91–272]	*<0.001*
Postmenopausal^a^	176 (14.6%)	78 (9.8%)	98 (23.9%)	*<0.001*
BMI (kg/m^2^)	25.3 ± 4.6	25.7 ± 4.8	24.8 ± 4.1	*0.003*
Current smoking	85 (7.0%)	46 (5.7%)	39 (9.4%)	*0.024*
Positive CVD family history^a^	723 (59.8%)	493 (61.9%)	230 (55.7%)	*0.041*
Diabetes Mellitus	13 (1.1%)	12 (1.5%)	1 (0.2%)	0.072
Hypertension	143 (11.8%)	114 (14.2%)	29 (7.0%)	*<0.001*
Antihypertensive drugs use	113 (9.3%)	90 (11.2%)	23 (5.6%)	*0.001*
Multivitamin use	331 (27.2%)	205 (25.5%)	126 (30.4%)	0.077
Glucose level (mmol/L)^a^	5.1 ± 0.8	5.2 ± 0.9	5.0 ± 0.5	*0.001*
Systolic BP (mmHg)^a^	113 [107–122]	115 [108–123]	111 [105–118]	*<0.001*
Diastolic BP (mmHg)^a^	71 [66–77]	73 [67–79]	69 [64–74]	*<0.001*
MAP (mmHg)^a^	87 [82–94]	89 [83–96]	84 [79–90]	*<0.001*
Baseline brachial artery diameter (mm)	3.53 ± 0.44	3.50 ± 0.43	3.60 ± 0.46	*<0.001*
Absolute FMD (mm)	0.14 [0.09–0.21]	0.14 [0.09–0.20]	0.14 [0.09–0.21]	0.846
Relative FMD (%)	3.9 [2.5–5.9]	3.9 [2.5–5.9]	3.9 [2.4–5.9]	0.514
Allometric FMD (%)	4.0 [2.5–6.0]	4.0 [2.5–6.0]	4.0 [2.4–6.1]	0.514
Absolute NGMD (mm)	0.59 ± 0.18	0.59 ± 0.18	0.59 ± 0.19	0.921
Relative NGMD (%)	17.0 ± 5.5	17.1 ± 5.5	16.8 ± 5.5	0.260
Dilation (FMD) in proportion of maximal dilation (NGMD) (%)^a^	24.3 [14.5–35.8]	23.8 [14.4–35.8]	24.5 [14.6–35.9]	0.962

a Variable consisted few missing values (<2.0%), valid percentages are presented. Continuous variables are reported as mean ± standard deviation in case of normal distribution, otherwise as median [IQR]. Categorical variables are reported as number (%). Statistically significant p-values are presented in cursive.

PE, preeclampsia; BMI, body mass index; CVD, cardiovascular disease; BP, blood pressure; MAP, mean arterial pressure; FMD, flow-mediated-dilation; NGMD, nitroglycerine-mediated dilation.

The age of the study population ranged between 22 and 62 years. Women with a history of PE were on average 6 years younger, less often in postmenopausal state, and at a shorter postpartum interval compared to women with normotensive pregnancy. Moreover, BMI, fasting glucose levels, SBP and MAP were higher and DBP level was lower in women with a history of PE.

Prevalence of hypertension (14.2 vs. 7.0%, *p* < 0.001), antihypertensive drug use (11.2 vs. 5.6%, *p* = 0.001), positive CVD family history (61.9 vs. 55.7%, *p* = 0.041) and smoking (5.7 vs. 9.4%, *p* = 0.024) were higher among women with a history of PE.

Baseline brachial artery diameter was lower in women with a history of PE than in those with normotensive pregnancies (p ≤ 0.008). No statistically significant differences in FMD (absolute, relative and allometric), NGMD (absolute and relative) and physiologic response as proportion of maximal dilatory capacity were found between both groups (Table 1).

### Association of age with FMD and NGMD

**Figure 2:**
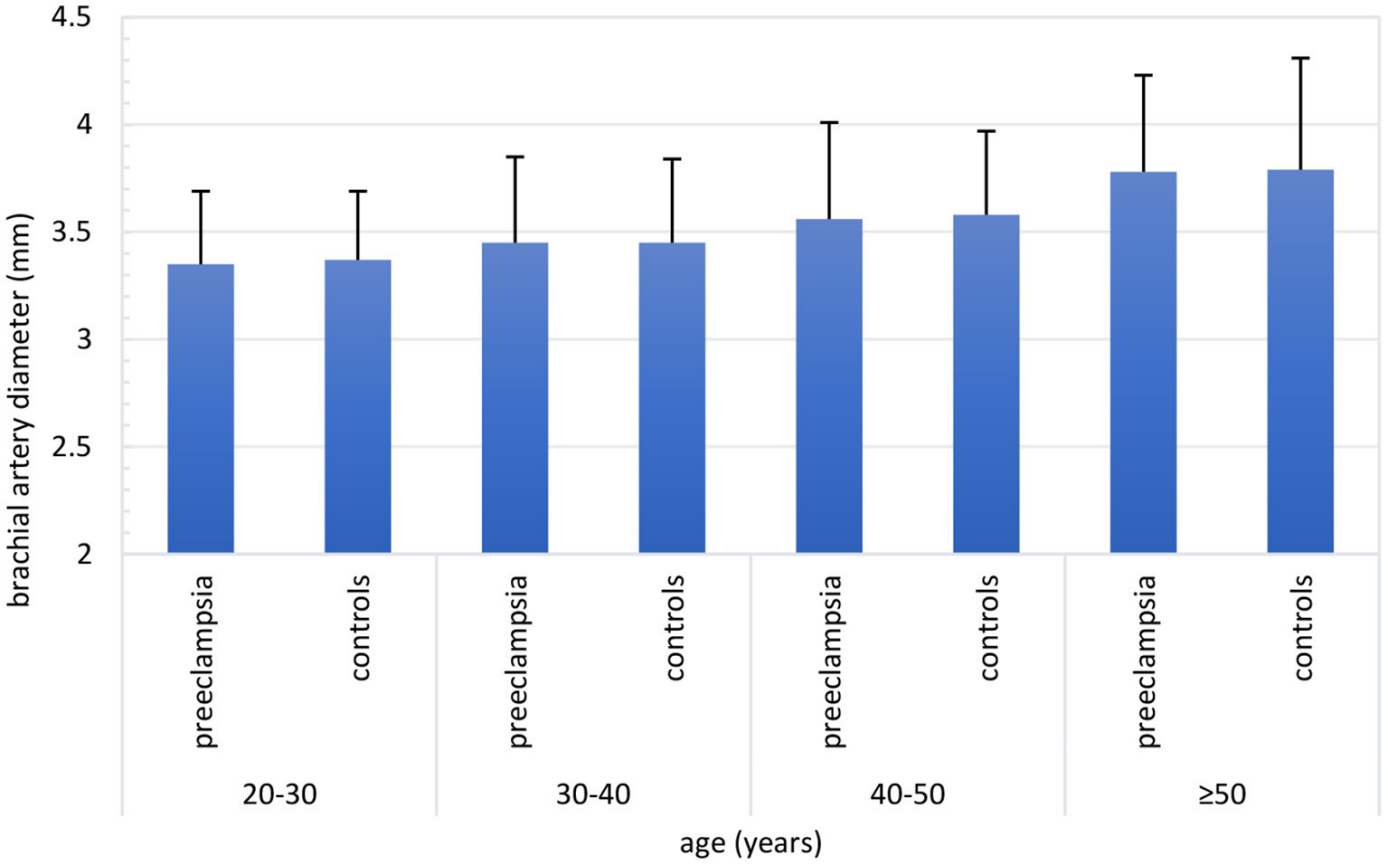
Brachial artery diameter (mm) across age categories stratified for women with a history of preeclampsia-complicated pregnancy (i.e. preeclampsia) and women with a history of normotensive pregnancy (i.e., controls). Both in women with and without a history of preeclampsia, brachial artery diameter increased with advancing age. Number of inclusions within groups: 20–30 years: preeclampsia *n* = 103, controls *n* = 16; 30–40 years: preeclampsia *n* = 392, controls *n* = 111; 40–50 years: preeclampsia *n* = 238, controls *n* = 159; ≥50 years: preeclampsia *n* = 70, controls *n* = 128.

**Figure 3:**
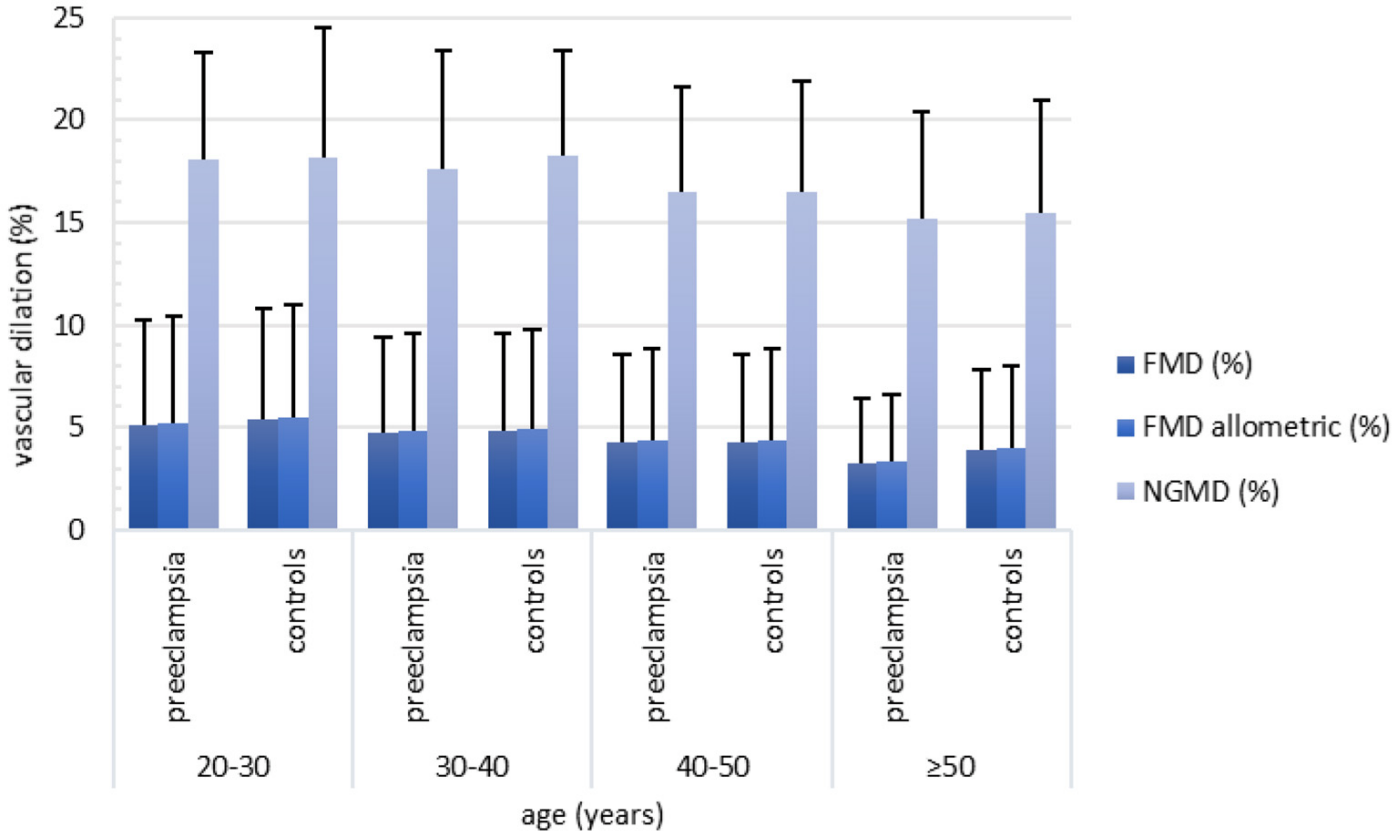
Relative FMD (%), allometric FMD (%) and NGMD (%) across age categories stratified for women with a history of preeclampsia-complicated pregnancy (i.e. preeclampsia) and women with a history of normotensive pregnancy (i.e. controls). Both in women with and without a history of preeclampsia, FMD (%), allometric FMD and NGMD (%) decreased with advancing age. Between women with preeclampsia and controls no clear differences were found with regard to FMD (%), allometric FMD (%) and NGMD (%). Number of inclusions within groups: 20–30 years: preeclampsia *n* = 103, controls *n* = 16; 30–40 years: preeclampsia *n* = 392, controls *n* = 111; 40–50 years: preeclampsia *n* = 238, controls *n* = 159, ≥50 years: preeclampsia *n* = 70, controls *n* = 128. FMD, flow-mediated-dilation; NGMD, nitroglycerine-mediated dilation.

Across age deciles, a statistically significantly increasing trend was found for brachial artery diameter, whereas for all parameters of FMD and NGMD a decreasing trend was found (Supplementary Table 2). These trends were similar for women with and without a history of PE (Figures 2, 3, Table 2). The dilation in response to stress stimulus (FMD) as proportion of maximal dilatory capacity (NGMD) decreased with aging in former preeclamptic women, but did not in controls (Supplementary Table 2). Though this remained not statistically significant in fully-adjusted regression models, the trend of a decreased ability to maximally dilate among former PE remained (Table 3).

**Table 2:** Trend effect of advancing age on FMD and NGMD.

**Measurement**	**20–30 yr (*n* = 119)**	**30–40 yr (*n* = 503)**	**40–50 yr (*n* = 397)**	**≥50 yr (*n* = 198)**	***p*-value of trend**
Brachial artery diameter (mm)	3.36 ± 0.34	3.45 ± 0.40	3.57 ± 0.43	3.79 ± 0.50	*<0.001*
FMD					
Absolute FMD (mm)	0.16 [0.11–0.22]	0.15 [0.09–0.21]	0.13 [0.09–0.20]	0.12 [0.07–0.18]	*<0.001*
Relative FMD (%)	4.7 [3.3–6.7]	4.4 [2.6–6.4]	3.6 [2.4–5.7]	3.1 [2.0–5.1]	*<0.001*
Allometric FMD (%)	4.8 [3.3–6.9]	4.5 [2.6–6.6]	3.7 [2.4–5.8]	3.1 [2.0–5.2]	*<0.001*
NGMD					
Absolute NGMD (mm)	0.60 ± 0.16	0.61 ± 0.18	0.58 ± 0.19	0.58 ± 0.20	*0.035*
Relative NGMD (%)	18.1 ± 5.4	17.8 ± 5.6	16.5 ± 5.2	15.4 ± 5.4	*<0.001*
Dilation (FMD) in proportion of maximal dilation (NGMD) (%)	27.7 [19.9–36.6]	24.4 [15.1–36.4]	23.9 [14.3–35.3]	20.5 [12.4–34.1]	*0.007*

Continuous variables are reported as mean ± standard deviation in case of normal distribution, otherwise as mean [IQR]. Categorical variables are reported as number (%). Statistically significant p-values are presented in cursive.

yrs, years; FMD, flow-mediated-dilation; NGMD, nitroglycerine-mediated dilation.

**Table 3:** Association of age and history of preeclampsia with brachial artery diameter, FMD and NGMD.

	**Brachial artery diameter (mm)**		**Absolute FMD (mm)**		**Relative FMD (%)**	
	β (95% CI), mm/10 yr	*p*-value	β (95% CI), mm/10 yr	*p*-value	β (95% CI), %/10 yr	*p*-value
**Unadjusted model**						
Age (in deciles)	0.16 (0.13–0.18)	*<0.001*	−0.01 (−0.02–−0.003)	*0.003*	−0.50 (−0.69–−0.32)	*<0.001*
History of PE	−0.01 (−0.07–0.04)	0.602	−0.008 (−0.02–0.004)	0.183	−0.15 (−0.49–0.19)	0.381
**Fully-adjusted model**						
Age (in deciles)	0.14 (0.10–0.17)	*<0.001*	−0.01 (−0.01–0.003)	0.060	−0.42 (−0.63–−0.21)	*<0.001*
History of PE	−0.06 (−0.12–−0.1)	*0.025*	−0.008 (−0.02–0.003)	0.155	−0.09 (−0.43–0.25)	0.598
	**Allometric FMD (%)**		**Absolute NGMD (mm)**		**Relative NGMD (%)**	
	β (95% CI), %/10 yr	*p*–value	β (95% CI), mm/10 yr	*p*–value	β (95% CI), %/10 yr	*p*–value
**Unadjusted model**						
Age (in deciles)	−0.52 (−0.72–−0.33)	*<0.001*	−0.02 (−0.04–−0.01)	*0.001*	−1.20 (−1.58–−0.81)	*<0.001*
History of PE	−0.16 (−0.51–0.20)	0.384	−0.01 (−0.04–0.01)	0.282	−0.36 (−1.05–0.34)	0.312
**Fully-adjusted model**						
Age (in deciles)	−0.44 (−0.66–−0.22)	*<0.001*	−0.02 (−0.03–−0.002)	*0.024*	−1.02 (−1.48–−0.56)	*<0.001*
History of PE	−0.09 (−0.44–0.26)	0.602	−0.001 (−0.03–0.02	0.951	0.09 (−0.63–0.81)	0.806
	**Dilation (FMD) as proportion of maximal dilation (NGMD) (%)**					
	β (95% CI), %/10 yr	*p*-value				
**Unadjusted model**						
Age (in deciles)	−1.47 (−2.67to −0.27)	*0.016*				
History of PE	−0.85 (−3.01 to 1.31)	0.441				
**Fully-adjusted model**						
Age (in deciles)	1.22 (−2.54 to 0.10)	0.071				
History of PE	−0.87 (−2.97 to 1.24)	0.417				

Fully-adjusted models adjusted for BMI, smoking, anti-hypertensive drug use, MAP, fasting glucose levels, menopausal state and family history of CVD. Regression on FMD and NGMD was additionally adjusted for stress stimulus and regression on absolute FMD and absolute FMD for baseline diameter. Statistically significant p-values are presented in cursive.

β, unstandardized regression coefficient; FMD, flow-mediated-dilation; NGMD, nitroglycerine-mediated dilation; 95% CI, 95% confidence interval.

In line, unadjusted linear regression models revealed a statistically significant association of advancing age with brachial artery diameter (regression coefficient (95% CI) = 0.16 mm/10 years (0.13–0.19 mm/10 years), *p* <0.001) and all parameters of FMD (absolute: −0.001 mm/10 years (95 % CI −0.002-−0.002 mm/10 years), relative: −0.48 mm/10 years (95% CI−0.65-−0.30 mm/10 years), allometric: −0.49 mm/10 years (95 % CI −0.68-−0.31 mm/10 years); *p* ≤ 0.007) and NGMD (absolute: −0.02 mm/10 years (95 % CI −0.03-−0.01), relative: −1.13 mm/10 years (95% CI −1.49-−0.77 mm/10 years); *p* ≤ 0.001) (Supplementary Table 3). This result remained similar after adjustment for confounders (*p* ≤ 0.018), with exception of the association of age with absolute FMD when adjusting for baseline diameter which became non-significant in multivariable analyses (regression coefficient = −0.01 mm/10 years (95% CI −0.01–0.003), *p*-value 0.060, Supplementary Table 3). If not adjusting for baseline, the effect of age on absolute FMD remained statistically significant in the multivariable analysis (regression coefficient = −0.01 mm/10 years (95 % CI −0.01-−0.001 mm/10 years), *p*-value 0.024).

### No interaction effect between PE and age on FMD and NGMD

For baseline brachial artery diameter, the interaction term between age and history of PE was not statistically significant, both in unadjusted and fully-adjusted linear regression models (interaction term *p* ≥ 0.588). Similar non-significant interactions were found for FMD (absolute, relative and allometric; *p*-values interaction ≥ 0.360), NGMD (absolute and relative; *p*-values interaction ≥ 0.443) and dilation as proportion of maximal dilatory capacity (≥ 0.224). These statistically non-significant interaction terms did not favor any stratification based on a history of PE.

Accordingly, we further investigated the independent effect of PE in all regression models. Following, for all measures of FMD and NGMD, no effect of a history of PE was found (*p* ≥ 0.170) (Table 3). For brachial artery diameter, however, after adjustment for age and other confounders the effect of a history of PE on brachial artery diameter was −0.06 mm (95% CI −0.12-−0.10 mm, *p* = 0.025) as compared to history of normotensive pregnancy, indicating smaller brachial arteries in women with PE (Table 3). The results on the association with age remained similar in these models as compared with the regression analyses described above (Supplementary Table 3).

### Sensitivity analyses

Sensitivity analyses on replacing age by postpartum interval did not provide different findings (data not shown), with exception of the confounder-adjusted association between postpartum interval and absolute NGMD which became non-significant (data not shown) in contrast to the reported result for age and absolute NGMD (Supplementary Table 3). Although the association between years postpartum and dilation as proportion of maximal dilation was not significant in univariable analyses, is was significant in multivariable analyses as in line with the analyses on age. Re-analyzing the data by replacing parameters for FMD and NGMD, as described in our methods section (i.e., replacing leading baseline by single-point baseline, and replacing relative FMD and relative NGMD by normalized relative FMD for stress stimulus instead of separate adjustment for stress stimulus), did also not alter the results (data not shown).

## Discussion

In this cross-sectional study we show that the age-related decline in brachial artery vasodilation and increase in diameter was independent of obstetric history, suggesting no additional (accelerating) effect of PE on the decline in endothelial function with aging. The age-related decline in both endothelium-dependent and –independent brachial artery vasodilation itself was significant even after adjusting for important confounding factors. PE itself was consistently associated with a smaller brachial artery diameter across ages, but did not affect observed FMD and NGMD.

### No accelerated age-effect on FMD and NGMD in former preeclamptic women

In agreement with previous studies, we show an independent decline in FMD and NGMD and increase in arterial diameter with advancing age (3, 23, 24). This may provide a valuable explanation for the increasing CVD risk with aging, especially as traditional risk factors do not completely explain the impact of age on CVD. We also showed that women with a history of PE have smaller brachial artery diameters at baseline, but showed similar age-related diameter and FMD/NGMD changes compared to women with a history of normotensive pregnancies. Physiologically, our study shows that the known PE-linked endothelial dysfunction does not relate to an accelerated endothelial decline with advancing age in former preeclamptic women. Clinically, the future cardiovascular risk after PE seems less likely attributable to an accelerated age-related decline in FMD or NGMD. The absence of an effect of NGMD in addition to the observed difference in diameter suggests smaller arteries in former preeclamptic women, although one would then expect to find differences in relative changes, which is not the case in our study.

Earlier studies found that FMD was diminished in preeclamptic women, even several weeks before diagnosis (6, 25). Also, in the first decade after PE, some studies demonstrated diminished FMD, whilst others did not find any difference in later time periods when compared to control groups (6, 26–30). A meta-analysis of Weissgerber et al. (6) demonstrated a decreased FMD only within the first 3 years after PE, after which no difference in FMD was found up to 10 years postpartum. Our study did not find any difference across all age groups. Severity and/or time of onset of PE may contribute to these conflicting results. Besides, endothelial (dys)function has many dimensions, of which FMD and NGMD are only two. Other endothelial functions might still be altered in former PE women (both independent as in interaction with age), for example, circulating markers that might represent early endothelial dysfunction, including soluble fms-like tyrosine kinase (sFlt-1) and high-sensitivity C-reactive protein, already are elevated after PE up and until 10 years postpartum (27, 31).

### Arterial aging in women

With advancing age, arteries demonstrate a systemic, gradual impairment in vascular endothelial function, which is likely due to functional, downregulation of vasodilator pathways (i.e., reduction in endothelial-derived nitric oxide (NO) bioavailability) and/or up-regulation of vasoconstrictor pathways (i.e., increased production of vasoconstrictors like endothelin-1) and structural vessel wall characteristics, amongst diameter and composition (3). The first functional change might specifically affect endothelium-dependent function (FMD), whereas the latter affects endothelium-independent function (NGMD). A lifetime exposure to (CV) risk factors and the susceptibility of individuals to the harmful consequences of these risk factors combined with aging itself may result in decreased arterial function (32). However, as aging and underlying progressive risk factors for disease are interrelated, it is a challenge to separate the so-called biological aging from aging-associated diseases.

The increase in brachial artery diameter over time may, at least partly, reflect a structural basis for an age-related reduction in dilatory capacity. With aging, smooth muscle cells undergo changes that may impact the vascular dilatory capability, such as changes in phenotype and senescence (33–35). As a result of these changes, elastic vessel properties are also altered, shifting the balance from elastin toward collagen. Consequently, arterial mechanical load due to blood pressure is more borne by the stiffer collagen in the arterial wall (3), at the expense of the relative dilatory capacity in response to endogenous or exogenous NO. The magnitude of (NO-mediated) vascular dilation also depends on the ability of the smooth muscle cells to relax which can be quantified by the maximum dilatory response following sublingual NG (3). Therefore, we interpret the age-related decline in FMD and NGMD as reflective of an altered smooth muscle phenotype, either by loss of bioavailability of, or sensitivity to NO, increase vasoconstriction activity, stiffer acting extra-cellular matrix and/or an already stretched vessel wall due to luminal enlargement (34, 36, 37). Unexpectedly, we did not find a fully-adjusted association between age and absolute FMD. Adjusting the absolute FMD by baseline diameter might, however, average out the age-related decline in dilation due to the age-related increase in baseline diameter or suggest that the age-related decline in function is mainly accountable to the age-related increase in brachial artery diameter.

With advancing age the most consistent structural changes include diameter enlargement (i.e., dilation), wall thickening (i.e., remodeling) and changes in wall content (e.g., loss of elastin), with related changes in elastic properties (3). We observed that the baseline diameter increases with advancing age, even after correcting for influencing factors, is in line with the age-related diameter enlargement described previously (23, 24). Interestingly, this increase in baseline diameter itself is independently associated with an increased risk of CVD (14, 38). Since the brachial artery is hardly prone to atherosclerosis, the perceived increase in baseline may more likely be related to age-related structural remodeling of the vessel wall rather than plaque formation. A history of PE was related to a smaller brachial artery diameter compared to normotensive gestation after correcting for confounders. This suggests a so far unknown vascular predisposition after hypertensive-pregnancy complications.

### Strengths and limitations

Several strengths and limitations merit attention in the interpretation of our results. Strengths of this study support internal validity of our findings and include (1) consideration of a longer age-interval than currently published studies and (2) our large sample size powering our study to detect even small effects. In addition, multiple operationalisations of FMD (absolute, relative, allometric) and NGMD (absolute, relative) were included in multivariable analyses and sensitivity analyses, which showed all similar results. This supported the robustness of our findings and further decreased information bias. The most commonly defined measure for FMD and NGMD is the percentage increase in diameter with respect to baseline. Some investigators argue that it is the absolute dilatory response that captures the endothelium-dependent dilatory capacity best, and hence, when using relative FMD, smaller vessels intrinsically show greater FMD (39). There are assumptions that correcting for this vessel diameter dependency by allometric scaling is the best operationalisation of FMD, though it did not affect our results. We found that allometrically-scaled FMD yielded similar results compared to FMD percentage increase, suggesting that differences in baseline vessel diameter did not fully explain age-related decline in FMD in our study population. Furthermore, the scaling exponent of 1.014 justified the use of FMD percentage increase in current study cohort.

Limitations of this study include the cross-sectional design which made it impossible for us to investigate causal pathways. This may have obscured an effect of PE on FMD and NGMD that might had been revealed with repeated measurements within individuals before and after pregnancy. Second, a decline in vascular function due to PE may only be apparent in a subgroup of women with a specific (CV) predisposition, which we, unfortunately could not distinguish in our study. Third, selection bias might have occurred in our control group, which might mitigated the observed effects. Women who perceived higher risk of CVD might have been more willing to participate in a CV study, in which personal advice on risk factors was given to participants. For example, the prevalence of a positive family history for CVD was higher within our study population than expected based on the general population, in which the prevalence ranges between 10 and 16% being depending on one's age (40). Finally, FMD and NGMD measurements are considered susceptible for methodological variability. However, in the hands of experienced sonographers and following a strict protocol, variations can be kept to a minimum (12, 41). Moreover, we used wall tracking software to improve reproducibility and data on the obstetric history was not available for the sonographer.

## Conclusion

This study shows that in young- to middle-aged women, vascular aging with respect to endothelium-dependent and -independent vessel dilation testing was similar in women after PE compared to women with a history of normotensive pregnancies, even though former preeclamptic women consistently have smaller brachial arteries. These findings suggest that the increased CV risk in the first decades after PE do not originate from an accelerated decline in endothelial function as measured by FMD or NGMD in conduit vessels. Different site (microvascular) and mode of endothelial action (hemostatic and inflammatory related integrity) might be involved, which remain subject for further investigation.

## Data availability statement

The raw data supporting the conclusions of this article will be made available by the authors, without undue reservation.

## Ethics statement

The studies involving human participants were reviewed and approved by Medical Ethics Committee of the Maastricht University Medical Centre (METC azM/UM). The patients/participants provided their written informed consent to participate in this study.

## Author contributions

EJ, VL, MS, and CG-D designed the content of current study. EJ performed the statistical analyses. EJ, MH, and VL wrote the manuscript. All authors critically reviewed the manuscript and approved the final version.

## Funding

This work was partially funded by the Dutch Heart Foundation (grant number 2013T084, Queen of Hearts study).

## Conflict of interest

The authors declare that the research was conducted in the absence of any commercial or financial relationships that could be construed as a potential conflict of interest.

## Publisher's note

All claims expressed in this article are solely those of the authors and do not necessarily represent those of their affiliated organizations, or those of the publisher, the editors and the reviewers. Any product that may be evaluated in this article, or claim that may be made by its manufacturer, is not guaranteed or endorsed by the publisher.
