# Supplementary material for: No accelerated arterial aging in relatively young women after preeclampsia as compared to normotensive pregnancy
Source: Front Cardiovasc Med. 2022 Jul 28;9:911603. doi: 10.3389/fcvm.2022.911603 (PMC9371444; doi:10.3389/fcvm.2022.911603)
Supplement: Supplementary file 1 [file Data_Sheet_1.docx]

***SUPPLEMENTARY FILE***

**NO ACCELERATED ARTERIAL AGEING IN RELATIVELY YOUNG WOMEN AFTER PREECLAMPSIA AS COMPARED WITH NORMOTENSIVE PREGNANCY – E.B.N.J Janssen et al. 2022**

**Table S1. Comparison between included and excluded women due to missing, incomplete or low-quality data on FMD and NGMD, and/or unknown fasting state.**

|  | Included  (n = 1,217) | Excluded  (n = 248) | p-value |
| --- | --- | --- | --- |
| Age (years) | 40.5 ± 8.6 | 40.6 ± 8.7 | 0.954 |
| History of PE | 803 (66.0%) | 169 (68.1%) | 0.555 |
| Parity | 2 [1–2] | 2 [1–2] | 0.809 |
| Months postpartum | 104 [28–198] | 100 [25–212] | 0.941 |
| Postmenopausal^a^ | 176 (14.6%) | 39 (15.8%) | 0.623 |
| BMI (kg/m^2^) | 25.3 ± 4.6 | 24.6 ± 4.5 | *0.018* |
| Current smoking | 85 (7.0%) | 20 (8.1%) | 0.589 |
| Positive CVD family history^a^ | 723 (59.8%) | 142 (57.7%) | 0.569 |
| Diabetes Mellitus | 13 (1.1%) | 1 (0.4%) | 0.487 |
| Hypertension | 143 (11.8%) | 33 (13.3%) | 0.520 |
| Antihypertensive drugs use | 113 (9.3%) | 27 (10.9%) | 0.409 |
| Multivitamin use | 331 (27.2%) | 64 (25.8%) | 0.695 |
| Glucose level (mmol/L) | 5.1 ± 0.8 | 5.1 ± 0.6 | 0.665 |
| Systolic BP (mmHg)^a^ | 113 [107–122] | 114 [107–123] | 0.266 |
| Diastolic BP (mmHg)^a^ | 71 [66–77] | 72 [66–79] | 0.345 |
| MAP (mmHg)^a^ | 87 [82–94] | 88 [82–97] | 0.447 |

^a^Variable consisted few missing values (<1.0%/≤ n = 10), valid percentages are presented. Continuous variables are reported as mean ± standard deviation in case of normal distribution, otherwise as median [IQR]. Categorical variables are reported as number (%). Statistically significant p-values are presented in cursive.

Abbreviations: PE, preeclampsia; BMI, body mass index; CVD, cardiovascular disease; BP, blood pressure; MAP, mean arterial pressure.

**Table S2. Trend ageing on FMD and NGMD in women a history of PE and normotensive pregnancy**

|  | Women with a history of PE  (n = 803) | Women without history of PE  (n = 414) | p-value |
| --- | --- | --- | --- |
| Brachial artery diameter (mm) |  |  |  |
| 20-30 years | 3.35 ± 0.34 | 3.37 ± 0.32 | 0.884 |
| 30-40 years | 3.45 ± 0.40 | 3.45 ± 0.39 | 0.962 |
| 40-50 years | 3.56 ± 0.45 | 3.58 ± 0.39 | 0.678 |
| ≥50 years | 3.78 ± 0.45 | 3.79 ± 0.52 | 0.851 |
| *age trend* | *<0.001* | *<0.001* |  |
| Absolute FMD (mm) |  |  |  |
| 20-30 years | 0.16 [0.11–0.22] | 0.17 [0.11–0.23] | 0.668 |
| 30-40 years | 0.15 [0.09–0.21] | 0.15 [0.09–0.22] | 0.491 |
| 40-50 years | 0.12 [0.08–0.20] | 0.13 [0.09–0.19] | 0.442 |
| ≥50 years | 0.10 [0.07–0.15] | 0.12 [0.08–0.20] | 0.056 |
| *age trend* | *<0.001* | *0.030* |  |
| Relative FMD (%) |  |  |  |
| 20-30 years | 4.6 [3.2–6.8] | 4.8 [3.7–6.6] | 0.654 |
| 30-40 years | 4.3 [2.6–6.4] | 4.6 [2.8–6.4] | 0.607 |
| 40-50 years | 3.6 [2.4–5.7] | 3.7 [2.7–5.7] | 0.646 |
| ≥50 years | 2.9 [1.7–4.1] | 3.4 [2.1–5.4] | 0.081 |
| *age trend* | *<0.001* | *0.001* |  |
| Allometric FMD (%) |  |  |  |
| 20-30 years | 4.7 [3.3–7.0] | 4.9 [3.7–6.8] | 0.654 |
| 30-40 years | 4.4 [2.6–6.6] | 4.7 [2.8–6.5] | 0.607 |
| 40-50 years | 3.7 [2.4–5.8] | 3.8 [2.7–5.8] | 0.646 |
| ≥50 years | 2.9 [1.7–4.2] | 3.4 [2.1–5.5] | 0.081 |
| *age trend* | *<0.001* | *0.001* |  |
| Absolute NGMD (mm) |  |  |  |
| 20-30 years | 0.60 ± 0.16 | 0.60 ± 0.18 | 0.988 |
| 30-40 years | 0.60 ± 0.16 | 0.62 ± 0.17 | 0.498 |
| 40-50 years | 0.58 ± 0.19 | 0.58 ± 0.19 | 0.850 |
| ≥50 years | 0.56 ± 0.16 | 0.59 ± 0.21 | 0.396 |
| *age trend* | *0.045* | *0.263* |  |
| Relative NGMD (%) |  |  |  |
| 20-30 years | 18.1 ± 5.2 | 18.2 ± 6.3 | 0.928 |
| 30-40 years | 18.1 ± 5.2 | 18.3 ± 5.1 | 0.283 |
| 40-50 years | 16.3 ± 5.1 | 16.5 ± 5.4 | 0.952 |
| ≥50 years | 15.2 ± 5.2 | 15.5 ± 5.5 | 0.690 |
| *age trend* | *<0.001* | *<0.001* |  |
| Dilation (FMD) in proportion of maximal dilation (NGMD) (%) | | |  |
| 20-30 years | 27.4 [19.9–36.5] | 29.5 [20.3–39.8] | 0.613 |
| 30-40 years | 24.5 [14.6–36.6] | 24.2 [16.3–35.5] | 0.671 |
| 40-50 years | 23.2 [13.4–34.9] | 25.5 [14.7–35.9] | 0.575 |
| ≥50 years | 17.6 [12.2–31.1] | 23.8 [12.4–35.2] | 0.103 |
| *age trend* | *0.003* | 0.502 |  |

Number of inclusions within groups: 20-30 years: preeclampsia n = 103, controls n = 16; 30-40 years: preeclampsia n = 392, controls n = 111; 40-50 years: preeclampsia n = 238, controls n = 159; ≥50 years: preeclampsia n = 70, controls n = 128.

Abbreviations: PE, preeclampsia; FMD, flow-mediated-dilation; NGMD, nitroglycerine-mediated dilation.

**Table S3. Association of age with FMD and NGMD**

|  | **Brachial artery diameter (mm)** | | **Absolute FMD (mm)** | | **Relative FMD (%)** | |
| --- | --- | --- | --- | --- | --- | --- |
|  | β (95% CI), mm/10yr | p-value | β (95% CI), mm/10yr | p-value | β (95% CI), %/10yr | p-value |
| **Unadjusted model** |  |  |  |  |  |  |
| Age (in deciles) | 0.16 (0.13–0.19) | *<0.001* | -0.01 (-0.02–-0.002) | *0.007* | -0.48 (-0.65–-0.30) | *<0.001* |
| **Fully-adjusted model** |  |  |  |  |  |  |
| Age (in deciles) | 0.15 (0.12–0.18) | *<0.001* | -0.01 (-0.01–0.002) | 0.130 | -0.40 (-0.60–-0.21) | *<0.001* |
|  | **Allometric FMD (%)** | | **Absolute NGMD (mm)** | | **Relative NGMD (%)** | |
|  | β (95% CI), %/10yr | p-value | β (95% CI), mm/10yr | p-value | β (95% CI), %/10yr | p-value |
| **Unadjusted model** |  |  |  |  |  |  |
| Age (in deciles) | -0.49 (-0.68–-0.31) | *<0.001* | -0.02 (-0.03–-0.01) | *0.001* | -1.13 (-1.49–-0.77) | *<0.001* |
| **Fully-adjusted model** |  |  |  |  |  |  |
| Age (in deciles) | -0.42 (-0.62–-0.21) | *<0.001* | -0.02 (-0.03 –-0.003) | *0.018* | -1.04 (-1.47–-0.61) | *<0.001* |
|  | **Dilation (FMD) as proportion of maximal dilation (NGMD) (%)** | | | | |  |
|  | β (95% CI), %/10yr | p-value |  |  |  |  |
| **Unadjusted model** |  |  |  |  |  |  |
| Age (in deciles) | -1.31 (-2.44 – -0.17) | *0.023* |  |  |  |  |
| **Fully-adjusted model** |  |  |  |  |  |  |
| Age (in deciles) | -1.04 (-2.28 – 0.21) | 0.103 |  |  |  |  |

Confounders adjusted for in fully-adjusted model: BMI, smoking, anti-hypertensive drug use, MAP, fasting glucose levels, menopausal state and family history of CVD. Regression on FMD and NGMD was additionally adjusted for stress stimulus and regression on absolute FMD and absolute FMD for baseline. Statistically significant p-values are presented in cursive.

Abbreviations: β, unstandardized regression coefficient; FMD, flow-mediated-dilation; NGMD, nitroglycerine-mediated dilation; 95% CI, 95% confidence interval.
